# Chondroitin Sulfate-Rich Extract of Skate Cartilage Attenuates Lipopolysaccharide-Induced Liver Damage in Mice

**DOI:** 10.3390/md15060178

**Published:** 2017-06-15

**Authors:** Yeong Ok Song, Mijeong Kim, Minji Woo, Jang-Mi Baek, Keon-Hee Kang, Sang-Ho Kim, Seong-Soo Roh, Chan Hum Park, Kap-Seop Jeong, Jeong-Sook Noh

**Affiliations:** 1 Department of Food Science and Nutrition and Kimchi Research Institute, Pusan National University, Busan 46241, Korea; yosong@pusan.ac.kr (Y.O.S.); mijeongkim@pusan.ac.kr (M.K.); woo07140@pusan.ac.kr (M.W.); 2 Yeongsan Skate Co. Ltd., Busan 48531, Korea; rose8653@naver.com (J.-M.B.); skate1438@naver.com (K.-H.K.); ssong6415@hanmail.net (S.-H.K.); 3 College of Korean Medicine, Daegu Haany University, Daegu 42158, Korea; ddede@dhu.ac.kr; 4 Department of Medicinal Crop Research, National Institute of Horticultural and Herbal Science, Rural Development Administration, Eumseong 55365, Korea; ptman123@korea.kr; 5 Department of Food Science & Nutrition, Tongmyong University, Busan 48520, Korea; ks0903@tu.ac.kr

**Keywords:** skate cartilage, chondroitin sulfate, inflammation, antioxidant enzyme, apoptosis, MAPK, SREBPs, lipopolysaccharide

## Abstract

The protective effects of a chondroitin sulfate-rich extract (CSE) from skate cartilage against lipopolysaccharide (LPS)-induced hepatic damage were investigated, and its mechanism of action was compared with that of chondroitin sulfate (CS) from shark cartilage. ICR mice were orally administrated 200 mg/kg body weight (BW) of CS or 400 mg/kg BW of CSE for 3 consecutive days, followed by a one-time intraperitoneal injection of LPS (20 mg/kg BW). The experimental groups were vehicle treatment without LPS injection (NC group), vehicle treatment with LPS injection (LPS group), CS pretreatment with LPS injection (CS group), and CSE pretreatment with LPS injection (CSE group). Hepatic antioxidant enzyme expression levels in the CS and CSE groups were increased relative to those in the LPS group. In LPS-insulted hepatic tissue, inflammatory factors were augmented relative to those in the NC group, but were significantly suppressed by pretreatment with CS or CSE. Moreover, CS and CSE alleviated the LPS-induced apoptotic factors and mitogen-activated protein kinase (MAPK). In addition, CS and CSE effectively decreased the serum lipid concentrations and downregulated hepatic sterol regulatory element-binding proteins expression. In conclusion, the skate CSE could protect against LPS-induced hepatic dyslipidemia, oxidative stress, inflammation, and apoptosis, probably through the regulation of MAPK signaling.

## 1. Introduction

**Figure 1:**
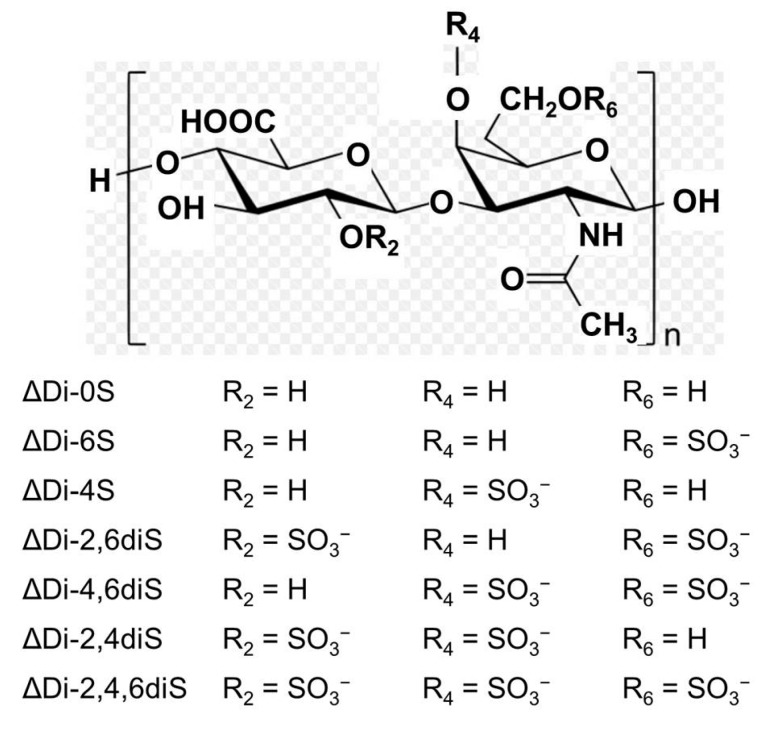
Structure of chondroitin sulfate.

Chondroitin sulfate (CS) is a glycosaminoglycan that is present in the extracellular matrix of animal tissue, especially in cartilages, skin, blood vessels, ligaments, and tendons, where it forms an essential component of proteoglycans (Figure 1) [1,2]. CS plays an important role in the elasticity and function of articular cartilage. In addition to these structural properties, CS exhibits a wide variety of biological functions due mainly to the presence of rare oversulfated building units in its domain structure that interact specifically with other molecules [3]. CS is currently recommended by the European League Against Rheumatism as a SYSADOA (symptomatic slow-acting drug for osteoarthritis) in the treatment of knee and hand osteoarthritis, based on research evidence and meta-analysis of numerous clinical studies [4]. The anti-inflammatory activity of CS has been suggested as a major function in the cellular system and human studies [5]. Consequently, the beneficial effects of CS have been recognized in chronic inflammatory diseases, such as atherosclerosis [6], inflammatory bowel disease [7], and psoriasis [8].

The liver is a major metabolic organ and plays a critical role in the defense of the body against bacteria and bacterial products, including lipopolysaccharides (LPSs) [9]. Impaired hepatic function, typically leading to hepatic encephalopathy and hydroperitoneum, is a common feature of the acute liver failure induced by LPS [10]. LPS stimulates Kupffer cells, the resident macrophages of the liver, to release toxic mediators, such as tumor necrosis factor-alpha (TNF-α), interleukins (ILs), platelet-activating factors, reactive oxygen species, and nitrogen species [11]. These proinflammatory mediators as well as the oxidative and nitrosative stress induced by LPS lead to hepatocyte death, which subsequently causes liver failure [12].

Members of the mitogen-activated protein kinase (MAPK) family, including p38 kinase, c-Jun N-terminal kinase (JNK), and extracellular signal regulated kinase (ERK), serve to regulate diverse cellular responses to extracellular stimuli, and modulate various cellular activities, including gene expression, mitosis, differentiation, and cell survival/apoptosis [13]. With regard to the regulation of MAPKs, it is well known that nuclear factor-kappaB (NF-κB) is a transcription factor involved in the expression of TNF-α and IL-1 [14]. The MAPK cascade plays a pivotal role in hepatocyte apoptosis and is considered to be an attractive therapeutic target. Therefore, an insight into the regulation of signaling pathways by MAPKs and NF-κB/activator protein-1 is indispensable for the future development of therapies to inhibit inflammation and apoptosis in acute liver injury.

The common skate (*Raja kenojei*) is a benthic animal living in the deep sea. Because only the meat portion is usually consumed, this organism yields approximately 30% of its weight in byproducts, consisting mostly of skin, bone and cartilage. These leftover products are a rich source of CS [15]. On the market, CS for drug purposes is produced from various sources, including cattle, pigs, chicken, or cartilaginous fish such as sharks [16]. In this study, the protective effects of the CS-rich extract (CSE) of skate cartilage against LPS-induced oxidative stress, inflammation, and apoptosis were studied, and its mechanism of action was compared with that of CS of shark cartilage origin. The mouse LPS-induced hepatic injury model used is a practical tool for the evaluation of nutraceutical or bioactive compounds that interfere with hepatic apoptosis and inflammatory liver injury.

## 2. Results

### 2.1. Effects of Skate CSE on LPS-Induced Decrease in Body and Liver Weights

As shown in Table 1, the final body weight of mice determined at 24 h after intraperitoneal LPS injection was significantly decreased in the LPS group compared to that in the NC group, although the initial body weights among all the experimental groups (37.7–38.0 g) were similar. There were significantly less body weight changes during 3 days in the CS and CSE groups than in the LPS group (*p* < 0.05). LPS treatment also significantly decreased the liver weight in the LPS group relative to that in the NC group, whereas CS and CSE treatments effectively protected against the reduction of liver weight (Table 1).

### 2.2. Effects of Skate CSE on LPS-Induced Peroxynitrite Radical Production, Serum Lipids, and Hepatic Function Parameters

**Figure 2:**
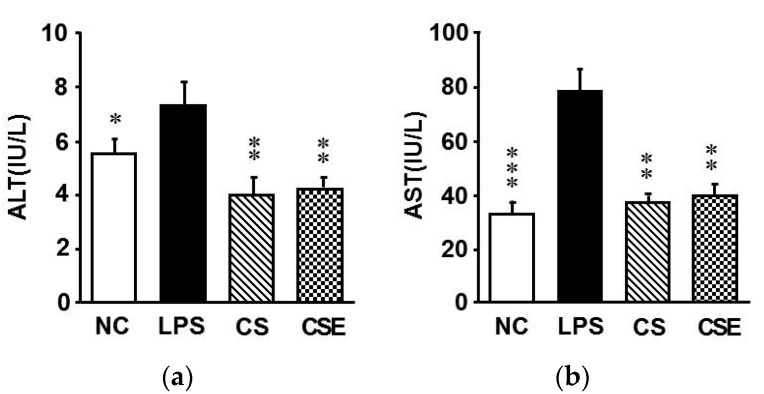
Serum transaminase activities. (**a**) Alanine transaminase; (**b**) Aspartate transaminase activities. NC, normal control mice; LPS, vehicle-treated LPS mice; CS, 200 mg/kg body weight chondroitin sulfate in LPS mice; CSE, 400 mg/kg body weight chondroitin sulfate preparation from skate cartilage in LPS mice. Data are the means ± S.E.M. (*n* = 8 mice per group). Significance: * *p* < 0.05, ** *p* < 0.01, *** *p* < 0.001 vs. vehicle-treated LPS mice.

As depicted in Table 2, the serum peroxynitrite (ONOO^−^) levels were 148.8% higher in the LPS group than in the NC group, whereas CS and CSE administration effectively decreased the levels by 30.6% and 29.8%, respectively, relative to that of the LPS group (*p* < 0.05). Serum concentrations of triglyceride and total cholesterol were significantly increased by the LPS injection (Table 2). In the CS and CSE groups, the serum triglyceride concentration was reduced by 35.7% and 47.0%, respectively, relative to that in the LPS group (*p* < 0.05). A significant decrease in the total cholesterol concentration was observed in both the CS and CSE groups (21.7% and 25.6%, respectively) compared with that in the LPS group (*p* < 0.05). LPS triggered a considerable increase in the alanine transaminase (ALT) and aspartate transaminase (AST) activities (Figure 2). However, compared with the levels in the LPS group, the ALT levels were 45.2% and 41.1% lower (*p* < 0.05) and the AST levels 52.9 and 48.9% lower (*p* < 0.05) in the CS and CSE groups, respectively.

### 2.3. Effects of Skate CSE on the Protein Expression of Antioxidant Enzymes in LPS-Induced Hepatic Tissue

**Figure 3:**
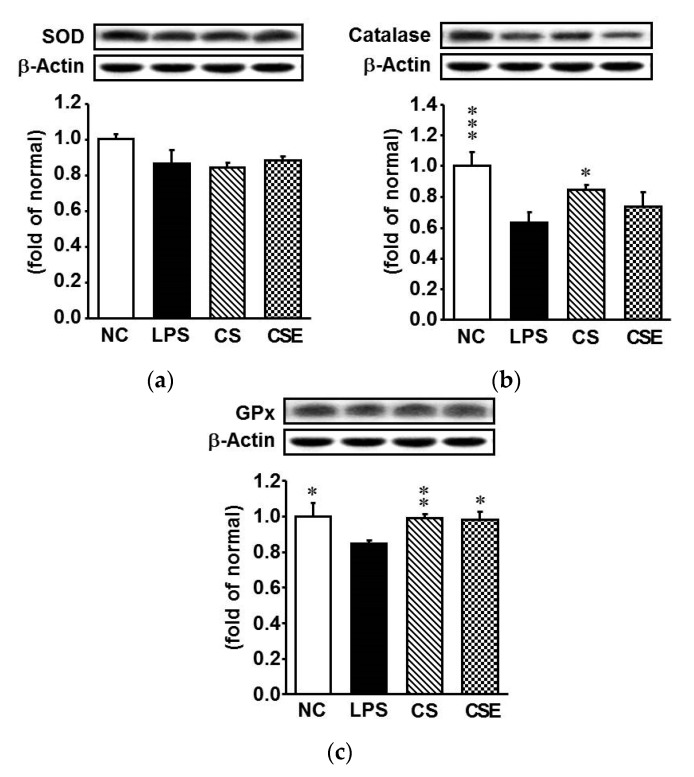
Protein expression of antioxidant enzymes in the hepatic tissue of lipopolysaccharide-induced ICR mice. (**a**) Superoxide dismutase; (**b**) Catalase; (**c**) Glutathione peroxidase. NC, normal control mice; LPS, vehicle-treated LPS mice; CS, 200 mg/kg body weight chondroitin sulfate in LPS mice; CSE, 400 mg/kg body weight chondroitin sulfate preparation from skate cartilage in LPS mice. Data are the means ± S.E.M. (*n* = 8 mice per group). Significance: * *p* < 0.05, ** *p* < 0.01, *** *p* < 0.001 vs. vehicle-treated LPS mice.

To determine the effects of CS and CSE on antioxidant enzymes such as superoxide dismutase (SOD), catalase, and glutathione peroxidase (GPx), western blot analysis was used to detect the hepatic protein levels in LPS-treated mice. Except for SOD, the protein expression levels of antioxidant enzymes were significantly downregulated at 24 h after LPS injection (Figure 3). As expected, pretreatment with CS or CSE markedly elevated the expression of catalase and GPx in the LPS-treated hepatic tissue, whereas that of GPx was increased by 18% and 21% in the CS and CSE groups, respectively, relative to the LPS group levels (*p* < 0.05). The augmentation of catalase and GPx expression by CS was higher than that by CSE.

### 2.4. Effects of Skate CSE on the Protein Expression of Proinflammatory Factors in LPS-Induced Hepatic Tissue

**Figure 4:**
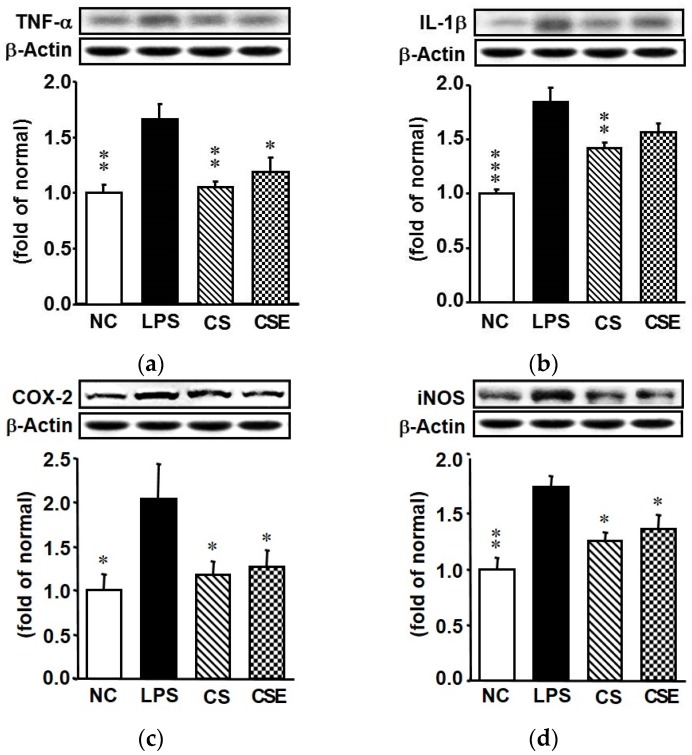
Protein expression of inflammatory mediators in the hepatic tissue of lipopolysaccharide-induced ICR mice. (**a**) Tumor necrosis factor-alpha; (**b**) Interleukin-1 beta; (**c**) Cyclooxygenase 2; (**d**) Inducible nitric oxide synthase. NC, normal control mice; LPS, vehicle-treated LPS mice; CS, 200 mg/kg body weight chondroitin sulfate in LPS mice; CSE, 400 mg/kg body weight chondroitin sulfate preparation from skate cartilage in LPS mice. Data are the means ± S.E.M. (*n* = 8 mice per group). Significance: * *p* < 0.05, ** *p* < 0.01, *** *p* < 0.001 vs. vehicle-treated LPS mice.

The effects of CS and CSE on LPS-induced changes in the hepatic proinflammatory TNF-α, IL-1β, cyclooxygenase (COX)-2, and inducible nitric oxide synthase (iNOS) proteins are shown in Figure 4. The LPS group showed a significant increase in expression of hepatic proinflammatory factors. On the other hand, the TNF-α, IL-1β, COX-2, and iNOS expression levels of the CS group were significantly lower (by 38.2%, 19.4%, 42.5%, and 27.8%, respectively) than those of the LPS group (*p* < 0.05). The TNF-α, COX-2, and iNOS expression levels of the CSE group were also significantly lower (by 29.4%, 37.9%, and 21.8%, respectively) than those of the LPS group (*p* < 0.05).

### 2.5. Effects of Skate CSE on the Protein Expression of Anti- and Pro-Apoptotic Mediators in LPS-Induced Hepatic Tissue

**Figure 5:**
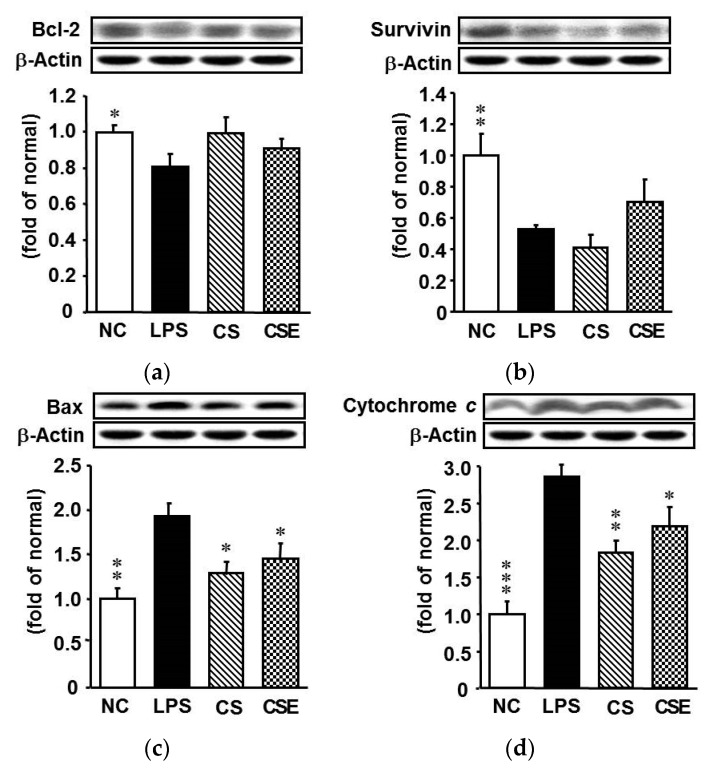
Protein expression of apoptosis-related factors in the hepatic tissue of lipopolysaccharide-induced ICR mice. (**a**) Bcl-2; (**b**) Survivin; (**c**) Bax; (**d**) Cytochrome *c*. NC, normal control mice; LPS, vehicle-treated LPS mice; CS, 200 mg/kg body weight chondroitin sulfate in LPS mice; CSE, 400 mg/kg body weight chondroitin sulfate preparation from skate cartilage in LPS mice. Data are the means ± S.E.M. (*n* = 8 mice per group). Significance: * *p* < 0.05, ** *p* < 0.01, *** *p* < 0.001 vs. vehicle-treated LPS mice.

LPS stimulated the hepatic apoptosis pathway in the mice, resulting in decreased anti-apoptotic Bcl-2 and survivin levels, and increased pro-apoptotic Bax and cytochrome c expression, compared with those of the NC group (Figure 5). The expression levels of Bax in the CS and CSE groups were reduced by 32.9% and 24.4%, respectively, compared with that in the LPS group (*p* < 0.05), whereas the cytochrome c protein expression levels were decreased significantly by 30.0% and 16.0%, respectively. There was no significant difference in the expression of survivin among experimental groups.

### 2.6. Effects of Skate CSE on the Protein Expression of Cell Signaling Transduction-Related Factors in LPS-Induced Hepatic Tissue

**Figure 6:**
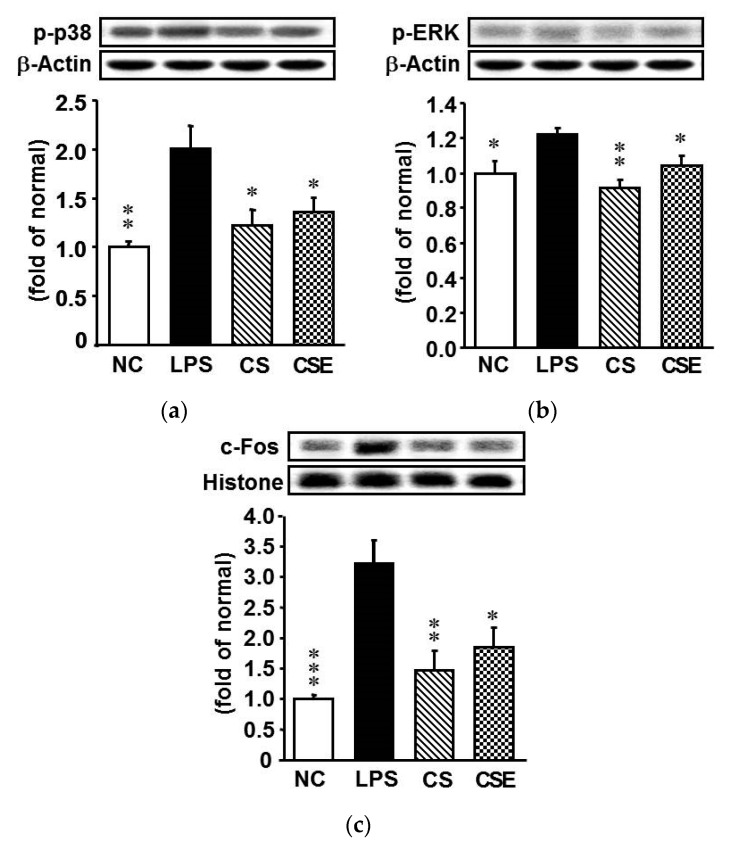
Protein expression of apoptosis-related factors in the hepatic tissue of lipopolysaccharide-induced ICR mice. (**a**) p-p38; (**b**) Phosphorylated extracellular signal regulated kinase; (**c**) c-Fos. NC, normal control mice; LPS, vehicle-treated LPS mice; CS, 200 mg/kg body weight chondroitin sulfate in LPS mice; CSE, 400 mg/kg body weight chondroitin sulfate preparation from skate cartilage in LPS mice. Data are the means ± S.E.M. (*n* = 8 mice per group). Significance: * *p* < 0.05, ** *p* < 0.01, *** *p* < 0.001 vs. vehicle-treated LPS mice.

Several important mechanisms of cell regulation involve signal transduction via MAPKs. To understand the signaling mechanisms of LPS-induced expression of hepatic inflammatory factors (TNF-α, IL-1β, COX-2, and iNOS), we determined the activation patterns for p38, ERK1/2, and c-Fos. LPS upregulated the phosphorylated forms (i.e., activated forms) of p38, ERK1/2, and c-Fos (Figure 6), but these effects were attenuated by the CS or CSE pretreatments. The p-p38, p-ERK, and c-Fos expression levels in the CS group were decreased by 43.6%, 24.0%, and 54.2%, respectively, and those in the CSE group by 39.1%, 16.0%, and 42.7%, respectively, compared with the levels in the LPS group (*p* < 0.05).

### 2.7. Effects of Skate CSE on the Protein Expression of Lipid Metabolism-Related Transcription Factors in LPS-Induced Hepatic Tissue

**Figure 7:**
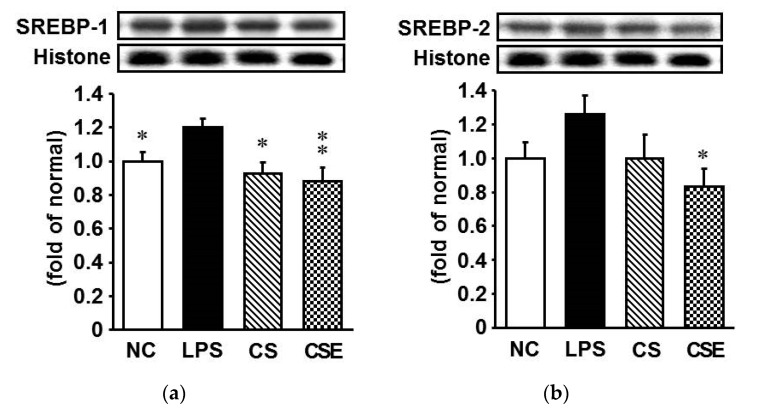
Protein expression of lipid metabolism-related transcription factors in the hepatic tissue of lipopolysaccharide-induced ICR mice. (**a**) Sterol regulatory element-binding protein (SREBP)-1; (**b**) SREBP-2. NC, normal control mice; LPS, vehicle-treated LPS mice; CS, 200 mg/kg body weight chondroitin sulfate in LPS mice; CSE, 400 mg/kg body weight chondroitin sulfate preparation from skate cartilage in LPS mice. Data are the means ± S.E.M. (*n* = 8 mice per group). Significance: * *p* < 0.05, ** *p* < 0.01 vs. vehicle-treated LPS mice.

To investigate the effect of LPS treatment on lipid metabolism in the liver, the levels of hepatic nuclear transcription factors such as sterol regulatory element-binding protein (SREBP)-1 and SREBP-2 were determined by western blot analysis. LPS treatment significantly increased the protein expression of SREBP-1 and SREBP-2, but this effect was attenuated by CS or CSE administration (Figure 7). SREBP-1 expression in the CS group was 20.8% lower than that in the LPS group (*p* < 0.05). In the CSE group, the SREBP-1 and SREBP-2 expression levels were 25.0% and 33.1% lower, respectively, than those in the LPS group (*p* < 0.05).

## 3. Discussion

In the present study, we investigated the inhibitory effect of skate CSEs on proinflammatory and apoptotic mediators, and explored the possible mechanism of action of the compound in the liver of LPS-injected mice. LPS, a bacterial endotoxin, causes serious damage in critical organs, such as the liver [17]. We found that CSE could protect mice against LPS-induced hepatic damage by decreasing oxidative stress and inflammatory cytokine and apoptotic marker expression, with concomitant upregulation of antioxidant enzymes and downregulation of the MAPK-dependent signaling pathway. Moreover, CSE attenuated LPS-induced hyperlipidemia by downregulating hepatic SREBPs levels. These beneficial effects of CSE were comparable to those of CS.

Under normal conditions, ALT is located in the cytoplasm, and AST is present in both the cytosolic and mitochondrial fractions of the liver. When the structure of the liver is damaged, ALT and AST are released into the circulatory system, resulting in increased activities in the blood [18]. In this study, CSE significantly alleviated the increased activity of AST and ALT mediated by LPS, revealing that skate CS has a preventative effect against LPS-induced liver damage.

Oxidative stress elevation is a common pathophysiological state observed in LPS-induced hepatic injury. Thus, augmentation of the antioxidant defense system in the body becomes necessary during infections, because redox imbalance is caused by the depletion of endogenous antioxidants, such as the antioxidant enzymes, and alteration of the glutathione redox status [19]. Numerous studies have tried to find new nutraceutical products with antioxidant and anti-inflammatory properties from natural sources, including plants or marine materials, to reverse and/or prevent hepatotoxicity [20,21,22]. CS demonstrated protective effects against CCl_4_-induced hepatotoxicity, attributed to its free radical-scavenging activity [23]. In addition, treatment with CS restored the activities of antioxidant enzymes in the liver of CCl_4_-injected [24] and ovariectomized rats [25]. These results are in line with those of our study. In the current study, the single injection of LPS elevated oxidative stress in the mice, whereupon the serum ONOO^−^ level increased but hepatic antioxidant enzymes such as catalase and GPx were significantly downregulated. CSE or CS administration to the mice prior to LPS treatment was able to significantly inhibit ONOO^−^ production and upregulate GPx.

In response to LPS, proinflammatory cytokines and enzymes are induced through NF-κB activation in the Kupffer cells, which are liver-resident macrophages [26]. Overproduction of proinflammatory cytokines has a detrimental effect, provoking an inflammatory reaction [27] that is dominated by the proinflammatory enzymes COX-2 and iNOS. Elevated inflammation in the liver causes liver damage [28]. Therefore, any substance that can attenuate the production of the proinflammatory cytokines and enzymes would be beneficial for delaying the progression of inflammation. In the present study, both CSE and CS were able to decrease the LPS-induced hepatic inflammation in the mice via downregulation of TNF-α, IL-1β, COX-2, and iNOS. It is apparent that CSE, like CS, has regulatory effects on the gene expression of proinflammatory cytokines and enzymes.

Cell survival is enhanced under the conditions of high Bcl-2 and low Bax expression. Previous studies have shown that LPS upregulates the pro-apoptotic Bax and downregulates the anti-apoptotic Bcl-2 levels [29]. However, CS pretreatment significantly attenuated 6-hydroxydopamine-induced apoptotic signals, including imbalance of the Bcl-2/Bax ratio, release of cytochrome c, and activation of caspase-9 and caspase-3 in a human neuroblastoma cell line [30]. Our current study has demonstrated that CSE, like CS, inhibits the LPS-induced increase of Bax and decrease of Bcl-2 expression in the liver of mice. Moreover, CSE and CS diminished the LPS-induced elevation of cytochrome c release, which is in line with previous findings [30,31]. CS could exert its anti-apoptotic effect by mitigating LPS-induced mitochondrial dysfunction.

To gain insight into the mechanism by which CSE or CS inhibits inflammation and apoptosis in the LPS-insulted liver of mice, we investigated MAPK signaling in the tissue. Numerous studies have shown that all MAPKs are activated by LPS [32,33,34]. The MAPKs (viz., p38 kinase, JNK, and ERK) react to extracellular stimuli and control a variety of cellular activities, including gene expression, mitosis, differentiation, and apoptosis [35]. In this study, we focused on MAPKs because they have been implicated in LPS-induced inflammation and apoptosis. We found that CSE and CS inhibited the phosphorylation of p38 and ERK. In IL-1β-stimulated cultured rabbit chondrocytes, CS decreased the IL-1β-induced phosphorylation of ERK1/2 and abrogated p38 MAPK phosphorylation [35]. The results from our study and other researchers suggest that inhibition of the phosphorylation of MAPKs by CSE or CS may contribute to the anti-inflammatory and anti-apoptotic effects in the liver of LPS-induced mice.

Hyperlipidemia frequently accompanies infectious and inflammatory diseases [36]. Several reports have demonstrated that LPS promotes hyperlipidemia via SREBP activation [37]. In this study, CSE and CS significantly reduced serum lipid levels as well as hepatic SREBP protein expression, which are in accordance with other reports [38]. CS reduced the serum triglyceride and total cholesterol levels in high-fat diet-fed rats [39]. Our results suggest that both CSE and CS could effectively ameliorate LPS-induced hyperlipidemia, likely through the inhibition of hepatic lipid synthesis, which is regulated by the nuclear transcription factor SREBP.

From Im et al. [40], the disaccharide composition of the CS obtained from skate cartilage is very similar to that from shark cartilage. Mammalian CS is generally composed of monosulfated disaccharides, such as ΔDi-4S (CS A) and ΔDi-6S (CS C), whereas marine CS contains oversulfated disaccharides, including ΔDi-2,6diS (CS D, shark cartilage), ΔDi-4,6diS (CS E, squid, salmon), and ΔDi-3,4diS (CS K, king crab) [41,42,43]. Although many commercial suppliers have obtained CS from mammalian species, which are more cost-effective than marine sources, there is increasing concern about the risk of animal epidemics, including bovine spongiform encephalopathy, foot-and-mouth disease, and bird influenzas. In addition, because of the restricted killing of sharks and high price of the raw material, CS from shark cartilage has a limited availability. Thus, the development of CS from the byproducts of other marine species, such as skate, is needed. In the present study, the liver-protecting effects of skate CSE were comparable to those of shark CS. Therefore, skate cartilage would be a good and readily available source for the development of CS for the functional food market.

## 4. Materials and Methods

### 4.1. Chondroitin Sulfate-Rich Extract of Skate Cartilage

The CSE was kindly provided by our collaborator (Yeongsan Skate Co., Ltd., Busan, Korea). The extract was prepared using the method of Nakano et al. [44], with slight modification. In brief, dried skate cartilage was hydrolyzed with alcalase (Protamex, Novozyme Co., Bagsvaerd, Denmark) at 50 °C for 4 h. The precipitated protein parts were removed by centrifugation (10,500× *g* for 10 min at 4 °C), and the supernatant was freeze-dried at −75 °C for preparation of the extract. The CS content of CSE was proximately 47.44% (*w*/*w*). Using the gel permeation chromatography, the molecular weight of CS in CSE was 284,671 Da. As a positive control, CS originated from shark cartilage was used (C4384; Sigma-Aldrich, St. Louis, MO, USA).

### 4.2. Animals and Experimental Protocol

Six-week-old male ICR mice were purchased from Samtako (Osan, Korea). The animals were maintained under a 12 h light/dark cycle, and housed at a controlled temperature (24 °C) and humidity (55% ± 5%). After 1-week acclimation, the mice were divided into 4 groups (*n* = 8) on the basis of their body weights: vehicle pretreatment without LPS injection (NC group), vehicle pretreatment with LPS injection (LPS group), CS pretreatment with LPS injection (CS group), and CSE pretreatment with LPS injection (CSE group). The vehicle-treated NC and LPS groups were given distilled water, whereas the CS and CSE groups were orally administered 200 mg/kg body weight (BW) of CS and 400 mg/kg BW of CSE, respectively, via a stomach tube, on a daily basis for 3 consecutive days. Then, the mice were intraperitoneally injected with LPS (20 mg/kg BW). The dose of CS was based on the previous report of Ha and Lee [23], whereas the dose of CSE was calculated from the above-mentioned CS content. After 24 h of LPS injection, blood was drawn from the heart. The liver was excised after perfusion with ice-cold PBS and kept at −80 °C until analysis. The animal experimental protocols were approved by the Institutional Animal Care and Animal Ethics Committee of Daegu Hanny University (Approval No. DHU2017-001) and the experiments were performed according to the Guidelines for Animal Experimentation.

### 4.3. Measurement of ONOO^−^ Level in the Serum

The ONOO^−^ level was evaluated using the method of Kooy et al. [45], with minor modifications. Serum was added to a rhodamine solution [50 mM sodium phosphate buffer, 90 mM NaCl, 5 mM diethylenetriaminepentaacetic acid, and dihydrorhodamine (DHR) 123], and then the fluorescence of rhodamine 123 (the reduced form of DHR 123) was measured at 485-nm excitation and 535-nm emission with a fluorescence plate reader at every 5 min for 30 min.

### 4.4. Preparation of Nuclear and Post-Nuclear Fractions

Nuclear protein extraction was performed according to the method of Komatsu [46]. In brief, liver tissues were homogenized with ice-cold lysis buffer containing 5 mM Tris-HCl (pH 7.5), 2 mM MgCl_2_, 15 mM CaCl_2_, and 1.5 M sucrose, and then 0.1 M dithiothreitol (DTT) and a protease inhibitor mixture solution were added. After centrifugation (10,500× *g* for 20 min at 4 °C), the pellet was suspended in extraction buffer containing 20 mM 2-[4-(2-hydroxyethyl)-1-piperazyl]ethanesulfonic acid (pH 7.9), 1.5 mM MgCl_2_, 0.42 M NaCl, 0.2 mM ethylenediaminetetraacetic acid, and 25% (*v*/*v*) glycerol, and then 0.1 M DTT and a protease inhibitor mixture solution were added. The mixture was placed on ice for 30 min. The nuclear fraction was obtained by centrifugation of the mixture at 20,500× *g* for 5 min at 4 °C. To obtain the post-nuclear fraction, the liver tissue was homogenized with ice-cold lysis buffer (pH 7.4) containing 137 mM NaCl, 20 mM Tris-HCl, 1% Tween 20, 10% glycerol, 1 mM phenylmethylsulfonyl fluoride, and a protease inhibitor mixture solution. The homogenate was then centrifuged at 2000× *g* for 10 min at 4 °C. The protein concentration in each fraction was determined using a Bio-Rad protein assay kit (Bio-Rad Laboratories, Hercules, CA, USA).

### 4.5. Immunoblotting Analysis

For the determination of c-Fos, SREBP-1, SREBP-2, and histone, 10 g of protein from each nuclear fraction was electrophoresed on a 12% sodium dodecyl sulfate polyacrylamide gel. The separated proteins were transferred to a nitrocellulose membrane, which was then blocked with 5% (*w*/*v*) skim milk solution for 1 h and thereafter incubated overnight with primary antibodies to c-Fos, SREBP-1, SREBP-2, and histone at 4 °C. After the blots had been washed, they were incubated with anti-rabbit or anti-mouse IgG horseradish peroxidase-conjugated secondary antibody for 1 h at 20 °C. Additionally, 10–15 g protein samples of each post-nuclear fraction of SOD, catalase, GPx, TNF-α, IL-1β, COX-2, iNOS, Bcl-2, survivin, Bax, cytochrome c, p-p38, p-ERK, and β-actin were electrophoresed by 8–15% SDS-PAGE. Each antigen–antibody complex was visualized using ECL western blotting detection reagents and detected by chemiluminescence with Sensi-Q 2000 (Lugen Sci, Gyeonggi-do, Korea). Band densities were determined using ATTO Densitograph Software (ATTO Corporation, Tokyo, Japan) and quantified as the ratio to histone or β-actin. The presented protein levels of each experimental group are relative to those of the normal mice.

### 4.6. Statistical Analysis

Eight samples per group all used for the examination of blood analysis and western blotting (*n* = 8, respectively) and each experiment was performed 3 times. Data are expressed as the mean ± S.E.M. Significance was assessed by one-way analysis of variance, followed by Dunnett’s multiple comparison test (SPSS 11.5.1 for Windows, 2002; SPSS, Inc., Chicago, IL, USA). Values of *p* < 0.05 were considered significant.

## 5. Conclusions

Our data showed that skate CS exhibited a remarkable protective effect against LPS-induced hepatic oxidative stress, inflammation, and apoptosis, probably via downregulation of MAPK signaling, and its activities were similar to those of shark CS.

## Figures and Tables

**Table 1:** Body weight and liver weight.

Group	Body Weight			Liver Weight (g/100 mg Body Weight)
	Initial (g)	Final (g)	Change (g/3 Days)	
NC	38.0 ± 0.5	40.2 ± 0.6 ***	1.9 ± 0.3 ***	8.1 ± 0.3 ***
LPS	37.8 ± 0.6	33.3 ± 0.7	−3.9 ± 0.4	5.6 ± 0.1
CS	37.7 ± 0.5	34.4 ± 0.5	−2.8 ± 0.3 *	7.2 ± 0.2 ***
CSE	37.7 ± 0.6	35.0 ± 0.5	−3.0 ± 0.1 *	6.6 ± 0.4 *

NC, normal control mice; LPS, vehicle-treated LPS mice; CS, 200 mg/kg body weight chondroitin sulfate in LPS mice; CSE, 400 mg/kg body weight chondroitin sulfate preparation from skate cartilage in LPS mice. Data are the means ± S.E.M. (*n* = 8 mice per group). Significance: * *p* < 0.05, *** *p* < 0.001 vs. vehicle-treated LPS mice.

**Table 2:** Serum biochemical analyses.

Group	Peroxynitrite (Fluorescence/mL)	Triglyceride (mg/dL)	Total Cholesterol (mg/dL)
NC	93.5 ± 1.5 **	166.4 ± 13.9 *	86.6 ± 2.7 *
LPS	139.1 ± 13.0	236.0 ± 27.6	94.8 ± 1.8
CS	96.6 ± 2.8 *	151.8 ± 9.6 *	74.2 ± 3.8 **
CSE	97.7 ± 3.7 *	125.1 ± 13.4 **	70.5 ± 5.3 **

NC, normal control mice; LPS, vehicle-treated LPS mice; CS, 200 mg/kg body weight chondroitin sulfate in LPS mice; CSE, 400 mg/kg body weight chondroitin sulfate preparation from skate cartilage in LPS mice. Data are the means ± S.E.M. (*n* = 8 mice per group). Significance: * *p* < 0.05, ** *p* < 0.01 vs. vehicle-treated LPS mice.
